# Obstinate Hæmorrhage Following the Extraction of a Tooth

**Published:** 1844-11-16

**Authors:** 


OBSTINATE HÆMORRHAGE FOLLOWING THE EXTRACTION OF A TOOTH.

Dr. J. Hartman states in the Neumeister's Repertorium, that a man of robust and vigorous constitution, who had ten years previously had an incisor tooth in the lower jaw extracted, followed by obstinate hæmorrhage, had another incisor tooth of the same jaw removed, shortly after which profuse hemorrhage occurred, and continued in spite of medical treatment, for five days, when he was conveyed in a state of great exhaustion to the general hospital at Vienna, where Dr. Bataffa, surgeon in chief of the district, determined to have recourse without delay to compression, which he practised in the following manner:—he prepared a cone, made with wax, the thickness of which corresponded with the diameter of the alveolus; this he soaked in a styptic mixture and then introduced it into the cavity. He afterwards applied two flat pieces, prepared from the same mass, of sufficient extent to cover over the alveolar orifice, and then placed over the whole a square plate, maintaining it *in situ* by a narrow bandage, conducted transversely in the direction of the angles of the mouth, and inclined downwards to the chin. In this place the bandage was drawn towards the occiput, in a line with the vertex, and terminated by being carried round the forehead. The patient was enjoined to preserve absolute repose of mind and body. A potion was prescribed for him, composed of barley-water, acidulated with *Haller's acid elixir*, and administered through a funnel. The hæmorrhage was arrested, and the patient rapidly regained his strength. After the third day the apparatus was removed without a return of the bleeding, and he left the hospital on the sixth day, cured.—*Ibid.*

